# 24-Hour ICH Score Is a Better Predictor of Outcome than Admission ICH Score

**DOI:** 10.1155/2013/605286

**Published:** 2013

**Authors:** Aimee M. Aysenne, Karen C. Albright, Tiffany Mathias, Tiffany R. Chang, Amelia K. Boehme, T. Mark Beasley, Sheryl Martin-Schild

**Affiliations:** 1 Stroke Program, Department of Neurology, Tulane University Hospital, 1440 Canal Street, TB-52, Suite 1000, New Orleans, LA 70112-2715, USA; 2 Neurovascular Service and Neurocritical Care, Department of Neurology, University of California, San Francisco, CA 94143, USA; 3 Health Services and Outcomes Research Center for Outcome and Effectiveness Research and Education (COERE), University of Alabama at Birmingham, Birmingham, AL 35249, USA; 4 Center of Excellence in Comparative Effectiveness Research for Eliminating Disparities (CERED), Minority Health & Health Disparities Research Center (MHRC), University of Alabama at Birmingham, Birmingham, AL 35249, USA; 5 School of Public Health, Department of Epidemiology, University of Alabama at Birmingham, Birmingham, AL 35249, USA; 6 Division of Neurosciences Critical Care, Department of Anesthesia Critical Care Medicine, Johns Hopkins University, Baltimore, MD 21287, USA; 7 School of Medicine, Department of Neurology, University of Alabama at Birmingham, Birmingham, AL 35249, USA; 8 Department of Biostatistics, School of Public Health, University of Alabama at Birmingham, Birmingham, AL 35294, USA

## Abstract

**Background:**

The ICH score is a validated tool for predicting 30-day morbidity and mortality in patients with intracerebral hemorrhage.

**Aims and/or Hypothesis:**

The aim of this study is to determine if the ICH score calculated 24 hours after admission is a better predictor of mortality than the ICH score calculated on admission.

**Methods:**

Patients presenting to our center with ICH from 7/08-12/10 were retrospectively identified from our prospective stroke registry. ICH scores were calculated based on initial Glasgow coma scale (GCS) and emergent head computed tomography (CT) on initial presentation and were recalculated after 24 hours.

**Results:**

A total of 91 patients out of 121 had complete data for admission and 24-hour ICH score. The ICH score changed in 38% from baseline to 24 hours. After adjusting for age, NIHSS on admission, and glucose, ICH score at 24 hours was a significant, independent predictor of mortality (OR = 2.71, 95% CI 1–19–6.20, and *P* = 0.018), but ICH score on admission was not (OR = 2.14, 95% CI 0.88-5.24, and *P* = 0.095).

**Conclusion:**

Early determination of the ICH score may incorrectly estimate the severity and expected outcome after ICH. Calculations of the ICH score 24 hours after admission will better predict early outcomes.

## 1. Introduction

The intracerebral hemorrhage (ICH) score was developed as a predictive tool for mortality at thirty days after hemorrhagic stroke [1]. The ICH score is a 6-point calculation based on five clinical indicators: age > 80 years, Glasgow coma scale (GCS), volume of hematoma on baseline CT scan, location (infratentorial or supratentorial), and the presence of intraventricular extension. The ICH score has also been validated for 30-day and one-year functional outcome in additional studies [2, 3]. In these studies the GCS was measured at the time of admission to the intensive care unit (ICU) or to the operating room regardless of the time of onset of symptoms.

Almost 40% of patients with brain imaging obtained in the first 3 hours after onset of symptoms of ICH experience hematoma expansion and this is highly associated with neurological deterioration [4]. Recent studies show a strong association between contrast extravasation on computed tomography angiography (CTA) and hematoma expansion and worse outcome [5].

## 2. Aims and/or Hypothesis

We hypothesize that, due to the dynamic nature of early ICH, with high risk of hematoma expansion, new IVH, and decreasing level of consciousness, a delayed measure of the ICH score would be more useful in predicting outcomes. In our center, the ICH score is calculated once all five clinical indicators are available, which is typical at the time when the baseline CT is performed, rather than when the patient went to the OR or neurological intensive care unit (ICU). We sought to determine the reliability of the ICH score calculated in the first hour of arrival compared to the reliability of the ICH score calculated 24 hours later.

## 3. Methods

A retrospective chart review of all patients admitted with ICH from July 2008 to December 2010 was performed. Patients included in this study were spontaneous, nontraumatic ICH with no structural underlying defect. Patients without the variables needed to calculate the admission and 24-hour ICH scores were excluded. ICH volume was calculated using the previously published ABC/2 method [6-8]. Initial ICH scores were calculated based on admission head CT and GCS on presentation to the hospital. Repeat ICH scores were calculated using follow-up imaging and GCS within 24 ± 1 hours of admission [1]. We compared outcomes using admission ICH score versus 24-hour ICH score. Our primary outcome of interest was poor functional outcome (mRS 5-6) at the time of hospital discharge. Our secondary outcome of interest was all-cause in-hospital mortality. Crude and adjusted logistic regression models were used to assess poor functional outcome and mortality using admission ICH score and 24-hour ICH score as predictors. The adjusted logistic regression models accounted for covariates previously shown to be confounders (age, NIHSS on admission, and glucose).

## 4. Results

Of the 119 patients who were eligible, 89 met inclusion criteria. Fourteen patients had mild stroke or had subacute presentations (median baseline ICH score 0) and were either not monitored in the neurological ICU or with routine follow-up CT. Fifteen patients had catastrophic stroke and either died prior to 24 hours or did not have follow-up CT (median baseline ICH score 3) within the specified timeframe. One patient was in the OR for evacuation and did not have GCS available during the specified timeframe. Table 1 provides a synopsis of the baseline demographics of the patients included in this study.

While the initial ICH score ranged from 0 to 3 (median 1), the 24-hour ICH score ranged from 0 to 5 (median 1). On admission, 21 (23.5%) patients had an ICH score of 0,33 (37%) patients had an ICH score of 1,19 (21.3%) patients had an ICH score of 2, and 16 (18%) patients had an ICH score of 3. No patients included in this study had an admission ICH score of 4, 5, or 6.

**Figure 1:**
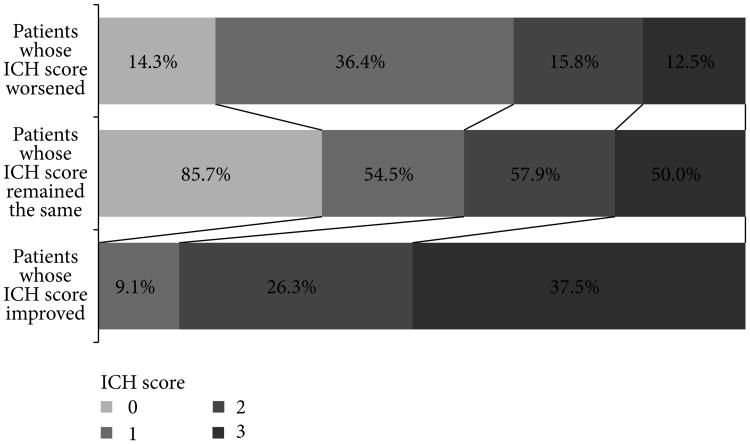
Differences in admission and 24-hour ICH score.

The distribution of ICH scores and frequencies of worsened, improved, or unchanged ICH score at 24 hours are presented in Table 2. Thirty-six percent of patients with ICH score of 1 on admission worsened on the 24-hour score while 38% of patients with ICH score of 3 on admission improved on the 24-hour score. The ICH score changed in the first 24 hours for 38% of patients with spontaneous ICH (Figure 1).

In the crude model, the odds ratio for poor functional outcome (OR = 5.975, 95% CI, and *P* < 0.0001) and death (OR = 3.562, 95% CI, and *P* < 0.0001) based on the 24-hour ICH score were greater than the admission ICH score odds ratios for both poor functional outcome (OR = 3.369, 95% CI, and *P* < 0.001) and death (OR = 2.933, 95% CI, and *P* = 0.001). After adjusting for age, baseline NIHSS, and admission glucose, baseline ICH score was not an independent predictor of poor functional outcome (OR = 1.904, 95% CI 0.937–3.869, and *P* = 0.075) or death (OR = 1.580, 95% CI 0.684–3.649, and *P* = 0.284). In the adjusted model, 24-hour ICH score was an independent predictor of poor functional outcome (OR = 4.672, 95% CI 1.996–10.939, and *P* < 0.0001) and death (OR = 2.712, 95% CI 1.187–6.195, and *P* = 0.018) (Table 3). It was a worsening in the GCS that resulted in worsening in ICH score at 24 hours in most cases (*n* = 14), followed by volume category increase (*n* = 5) and development of IVH (*n* = 3). Similarly, among patients with improved ICH score at 24 hours, the GCS improved in the majority of cases (*n* = 8) and volume reduced after evacuation in the others (*n* = 4).

## 5. Discussion

Our study found that 24-hour ICH score was better predictor of short-term functional outcome and mortality than initial ICH score. More than one-third of patients had a shif in the ICH score at 24 hours with similar proportions of patients with low scores worsening and high scores improving, mostly related to changes in GCS category. Although the ICH score was shown to be predictive of outcome, the exact time the ICH scores were calculated from the time of symptom onset is not known. Given that components of the ICH score are dynamic soon after ICH onset, particularly with hematoma expansion or interventions and procedures actively changing ICH volume, GCS, and IVH, the utility of using a predictive scoring system at 24-hours instead of on admission to predict outcomes increases the predictive value of the scoring system. The 24 hour ICH score is more precise. During this hyperacute phase, the ICH score on admission may underestimate the severity of the stroke since an ICH score can change rapidly due to decline in GCS or hematoma expansion. Likewise, early interventional therapies may improve the score with hematoma evacuation, hemicraniectomy, and appropriate management of intracranial pressure or intraventricular tissue plasminogen activator. Our data supports that early prognostication of outcomes should be avoided when possible, and using an ICH score calculated at a later time during the ICH admission is a better indicator of patient outcomes, after the hematoma has been stabilized and definitive therapies have been provided.

These findings further support how crucial the treatment of a patient within the first few hours after development of ICH is, particularly for interventions focused on prevention of hematoma expansion by blood pressure control, consideration of hemostatic agents and evacuation of hematoma or hemicraniectomy when appropriate, and prevention of complications such as aspiration. The ICH score is not a predictor of the outcome of an individual patient but a population-based guide that can aid in predicting the potential outcomes for ICH patients. These results provide continued evidence that treating physicians should remain vigilant with patient care for the ICH patient and provide aggressive measures until the course is declared.

Our study was limited by small sample size and our ability to adjust for other factors, such as blood pressure control and interventional procedures, both of which could affect the outcome. Evaluation of patients with more severe hemorrhages (ICH scores of 4, 5, and 6) was not possible due to unavailable data including follow-up ICH volume and/or GCS, raising the question of a self-fulfilling prophecy with early withdrawal of aggressive measures. This is, however, a study of early evolution of ICH score among patients with a reasonable chance of survival. It is possible that some patients with baseline ICH scores of 4 or 5 could be converted to lesser ICH scores, but we were unable to assess this in our population due to the information not being available. Further investigation in a larger sample is needed to validate our findings. A prospective study of the impact of time from symptom onset to calculation of the ICH score may be warranted to determine when, after ICH, the ICH score becomes reliable.

## Figures and Tables

**Table 1:** Demographics information.

	*N* = 89
Age, mean (sd)	58.07 (1.4)
Male, %	46 (51.7%)
Black, %	67 (75.3%)
History of HTN, %	71 (79.8%)
Antihypertensive medications, %	44 (49.4%)
On antithrombotics (AP/AC), %	32 (36%)
History of chronic daily alcohol use, %	23 (25.8%)
Positive urine tox screen, %	17 (19.1%)
ICH score on admission, median	1 (0–3)
Presenting GCS, median	14 (4–15)
First SBP, mean (sd)	189.7 (3.7)
First DBP, mean (sd)	111.2 (2.6)
Baseline NIHSS, median (range)	15 (0–40)
Glucose on admission, mean (sd)	144.65 (6.7)
ICH location	
Basal ganglia	41 (46.1%)
Talamus	18 (20.2%)
Pons	5 (5.6%)
Cerebellum	5 (5.6%)
Lobar	18 (20.2%)
Other	1 (1.1%)
ICH tentorial location	
Supratentorial	77 (86.5%)
Infratentorial	11 (12.4%)
To t a l	**88 (98.9%)**
IVH, %	42 (47.2%)
Hydrocephalus, %	35 (39.3%)
Edema on initial HCT, %	62 (69.7%)
Spot sign on CTA, %	10 (11.2%)
Dot sign present on CTA, %	16 (18%)
New IVH, %	6 (6.7%)
ICH expansion, %	42 (47.2%)
Evacuation, %	12 (13.5%)
EVD placed, %	28 (31.5%)
Did patient receive vitamin k? %	10 (11.2%)
Did patient receive FFP? %	14 (15.7%)
Did patient receive platelets? %	17 (19.1%)
Did patient receive NOVO7? %	4 (4.5%)
IVtPA, %	5 (5.6%)
In-hospital infection, %	33 (37.1%)
In-hospital DVT, %	2 (2.2%)
In-hospital UTI, %	24 (27%)
In-hospital bacteremia, %	12 (13.5%)
Best 24 hr GCS, median	13 (3–15)
Follow-up volume, median (range)	12.3 (0–402)
24-hour ICH score, median	1.0 (0–5)
Transfer patient, %	8 (9%)
Initial shift on HCT, median (range)	2.0 (0–17)
ICH initial volume, median (range)	12.8 (0–186)
ICH volume 24 hrs, median (range)	11 (0–402)
ICH volume growth in 24 hrs, median (range)	0 (-107-346)
Length of stay, median (range)	10 (2–86)
mRS on discharge, median (range)	4 (1–6)
Death, %	14 (15.7%)

**Table 2:** Baseline ICH score stratified by 24-hour ICH score status.

ICH score	ICH score of 0 (*N* = 21)	ICH score of 1 (*N* = 33)	ICH score of 2 (*N* = 19)	ICH score of 3 (*N* = 16)	*P* value*
Number of patients whose ICH score worsened	3 (14.3%)	12 (36.4%)	3 (15.8%)	2 (12.5%)	0.118
Number of patients whose ICH score remained the same	18 (85.7%)	18 (54.5%)	11 (57.9%)	8 (50.0%)	0.076
Number of patients whose ICH score improved	0	3 (9.1%)	5 (26.3%)	6 (37.5%)	0.006

* *P* value for comparison of each row.

**Table 3:** Logistic regression models.

	OR	95% CI	*P* value
Crude model predicting death			
Admission ICH score	2.933	1.510–5.699	0.001
24-hour ICH score	3.562	1.754-7.233	<0.0001
Crude model predicting poor mRS (5-6)			
Admission ICH score	3.369	1.934-5.869	<0.001
24-hour ICH score	5.975 2	.812-12.696	<0.0001
Adjusted model predicting death*			
Admission ICH score	1.580 0	.684–3.649	0.284
24-hour ICH score	2.712	1.187–6.195	0.018
Adjusted model predicting poor mRS (5-6)			
Admission ICH score	1.904	0.937–3.869	0.075
24-hour ICH score	4.672 1	.996-10.939	<0.0001

* Adjusting for age, baseline NIHSS, and glucose on admission.
